# 

**Published:** 1893-05-13

**Authors:** 


**OUR NEW OFFICES.**

428, Strand, W.C., will henceforth be the Office of this Journal.

**Notices of Births, Deaths, and Marriages,** are inserted in this column at a charge of 2s. 6d. for an announcement not exceeding four lines.

### NOTICE TO CORRESPONDENTS.

All MS., Letters, Books for Review, and other matters intended for the Editor should be addressed The Editor, The Lodge, Porchester Square, London, W.

The Editor cannot undertake to return rejected M.S., even when accompanied by stamped directed envelope.

**Notice to Advertisers and Subscribers.**

All Advertisements, Orders for Copies of The Hospital, and Business Communications should be addressed to The Publisher, *not to the Editor*, The Hospital, 428, Strand, W.C.

The Cost of Subscription, post free, is as follows:—Per week, 2½d.; per quarter, 2s. 6d.; per half-year, 5s.; per annum, 8s. 6d. Foreign:—Per annum, 10s. 8d.; payable, in all cases, in advance.

**"Burdett's Hospital Annual," 1893.**

Fourth year of publication. 700 pp. Boards, gilt lettering. A most complete guide to British, Colonial, Indian, and American Hospitals, Dispensaries, Nursing and Convalescent Institutions, and Asylums. Price 5s. Published by The Scientific Press (Limited), 428, Strand, W.C.

**NOTICE.—The Index for Vol. XIII. (ending March 1893) is on sale at the Office of this Journal, price 2½d., post free.**

### SCRAPS AND GLEANINGS.

It is proposed to erect an infectious diseases hospital at Malmesbury.

The numbers of new out-patients to the Bedford County Hospital for the quarter was reported at 2,421.

The editor of the Roumanian paper of anti-dynastic teaching has been ordered by the Government to leave the country.

The Queen has intimated, through Sir Henry Ponsonby, her intention to send a donation of £20 to the Richmond Hospital.

Leprosy exists to a considerable extent in the Transvaal, more than 50 Kaffir villages are reported infected by this disease.

It is stated that petroleum is a cure for diphtheria. Dr. Flanhaut, who made the discovery, has treated 40 cases with success.

Contributions of toys and picture-books are earnestly requested to furnish the large children's medical ward at the Leeds Infirmary.

The Mayor and Corporation of Rochester attended the Hospital Sunday Collection at the cathedral. The sum collected was £30.

Mr. George H. Strutt, of Bridge Hill, Belper, has given the munificent gift of £4,000 towards the building fund of the Derbyshire Royal Infirmary.

The Duke of Connaught presided at the 80th anniversary dinner of the London Orphan Asylum, donations to the extent of £2,957 were announced.

A concert, in aid of the funds of the Heatherdene Convalescent Home at Harrowgate, took place at the Victoria Hall, Harrowgate, on April 25th.

A bazaar and jumble sale was held recently in Northallerton Town Hall, in order to raise money for the sorely-needed building fund of the Northallerton Cottage Hospital.

There are to be new buildings at Paris for the Académie de Medécine. The site thus selected has previously been occupied by a large pawnbroking establishment.

In acknowledgment of the protests raised by the medical faculty at Havana, the Spanish Government has granted anew the right of the University to confer medical degrees.

The Society for Prevention of Cruelty to Animals has lost Lord Aberdare as its president. The Duke of York, at the invitation of the council, has consented to fill the vacant office.

The Erith and District Cottage Hospital has a balance of £19 on the year's working. The hospital has now attained its majority, and has entered on the twenty second year of its working.

The Treasurer's statement of the Stratton Cottage Hospital showed a balance from last year of £42. It was further stated that the number of patients received during the twelve months was 38.

The new wing at the Bristol Eye Infirmary was formally opened on April 20th, the Duchess of Beaufort presiding. The sum of £800 is still required to make up the £3,000 spent on this addition.

The Birmingham Skin and Lock Hospital held its twelfth annual meeting in April. The accounts show a surplus of £270 to the good. The number of cases treated during the year had been 4,469.

The London Volunteer Medical Staff have offered their services to the Metropolitan Asylums Board, to aid in conveying patients suffering from cholera to the various hospitals selected for their reception. The offer has been accepted.

A daily paper has recently stated that when Miss Ellen Terry is asked for her photograph, she turns the occasion into a means of contributing to hospitals, for she asks in these cases for a few pennies towards this object.

The Committee of the Salisbury Infirmary state in their report that the building improvement fund account is at present overdrawn at the bank by £1,525. the tender for the extension being higher than was originally anticipated.

A meeting of the Watford Rural Sanitary Authority was held in April to propose an isolation hospital for the district. The usual difficulty of selecting a suitable site gave rise to much discussion, and the matter is still under consideration.

Statistics go to prove that the early life of Italian children is somewhat more precarious than is that of other nations. In France, Belgium, and England, out of 1.000,000 children, 280,000 die before their tenth year; whilst in Italy the average is 480 000.

Application has been made to the Local Government Board by the Town Councils of Ramsgate and Margate and the Local Board of Broadstairs for a provisional order to form an united district for the purpose of providing an infectious diseases hospital.

The Duke of Portland has made a public appeal in the *Times* newspaper for funds towards the Metropolitan Hospital, Kingsland Road. Contributions are greatly needed, as 24 beds were closed a few weeks ago, the income of the hospital being insufficient for their support.

An excellent scheme has been carried into effect at Haverfordwest, whereby the town has been systematically canvassed by a committee of ladies collecting subscriptions for the Pembrokeshire Infirmary, the number of patients to the infirmary having much increased this last year.

The statement of accounts at the Carmarthenshire Infirmary shows an adverse balance of £6[illegible], being a reduction on the heavier one of last year. A slight falling off in contributions was referred to, but an increase of donations and collections. It is proposed to start a Hospital Saturday.

At the annual meeting of the subscribers to the Aberdeen Incurable Hospital, it was stated in the report that the average daily number of patients throughout the year was 61. The ordinary expenditure for the year amounted to £1,684, while the ordinary income was £750. Legacies had been received which met the deficiency, and had left a sum of £843.

The report of the Pendlebury Children's Hospital at Manchester states that 9,531 children have been treated in the year. The finance is, unfortunately, not in a flourishing condition. It is even hinted that if the income is not increased, the expenditure must be diminished by closing one of the wards.

### Medical Vacancies and Appointments.

**APPOINTMENTS.**

G. Armstrong, M.B., Ch.B.Melb., appointed Medical Superintendent to the Sydney Hospital, New South Wales. T. R. Bailey, M.D.Edin., reappointed Medical Officer of Health for Bilston. R. Barrett, M.D.Q.U.I., L.R.C.S.Edin., appointed Medical Officer for the Workhouse of the Macroom Union. W. B. Betenson, M.R.C.S.Eng., L.R.C.P.Lond., appointed Medical Officer for the seventh District of the Depwade Union. W. B. Bottomley, B.A., Ph.D., F.L.S., appointed Professor of Botany to King's College, London. J. A. Burland, L.R.C.P., L.R.C.S.I., appointed Medical Officer for the Prees District of the Wem Union. F. J. Butt, M.B., C.M.Edin., appointed Medical Officer for the North-West District of the Chester Union. W. F. Cleaver, L.R.C.P.Lond., M.R.C.S., appointed Medical Officer of Health to the Phillack Urban Sanitary District. E. O. Croft, M.R.C.S., L.R.C.P., appointed Honorary Demonstrator in Midwifery to the Medical Department of the Yorkshire College, Leeds. J. J. Curran, L.R.C.P.Edin., L.R.C.S.I., appointed Certifying Factory Surgeon for Killeagh, co. Cork. J. Donald, M.R.C.S., appointed Medical Officer for the Kingston Workhouse. T. M. Draper, L.R.C.P.Lond., M.R.C.S.Eng., appointed Medical Officer for the Wiggenhall District of the Downham Union. A. B. Duffin, M.D., F.R.C.P., appointed Joint Professor of Systematic Medicine to King's College, London, in conjunction with Professor Lionel Beale, F.R.S. S. Ellis, M.R.C.S., L.S.A., appointed Medical Officer for the third and sixth Districts, and Public Vaccinator for the third and fifth Districts of the Alderbury Union. W.K. Fyffe, M.B.Camb., M.R.C.P.Lond., appointed Pathologist to the City of London Hospital for Diseases of the Chest, Victoria Park. M. Grigor, L.R.C.P., L.R.C.S.Edin., appointed Medical Officer for the second Battersea District of the Wandsworth and Clapham Union. W. S. Griffith, M.A., M.B., B.C.Cantab., appointed Senior Resident Medical Officer, Royal Free Hospital, Gray's Inn Road. L. D. Heather, M.R.C.S., L.R.C.P., appointed House Surgeon to King's College Hospital. E. Henry, M.R.C.S., L.R.C.P., appointed Junior Resident Medical Officer, Royal Free Hospital, Gray's Inn Road. H. De Dreux Kunz, M.A., M.B. Edin., appointed Medical Officer to the Elgin Lodge of Oddfellows. T. C. Lawson, M.R.C.S.Eng., appointed Medical Officer for the fourth District of the St. Albans Union. C. E. M. Lowe, M.B.Vict., appointed Junior House Surgeon to the Royal Albert Edward Infirmary, Wigan. J. Lynass, M.B., B.Ch.R.U.I., appointed Resident Medical Officer of the Belfast Union Infirmary. G. MacDonald, M.D., appointed Assistant Physician to King's College Hospital, with special charge of Throat Department. G. H. Metcalfe, L.R.C.P.Lond., M.R.C.S.Eng., appointed Medical Officer for the fourth District of the Sudbury Union. H. C. Mooney, L.R.C.P., L.M., L.R.C.S.I., appointed Resident Medical Officer to the National Eye and Ear Infirmary, Dublin. J. D. Munro, M.B., C.M.Edin., appointed Senior House Surgeon to the Royal Albert Edward Infirmary, Wigan. Mary C. Murdoch, L.R.C.P., L.R.C.S.Edin., L.F.P. and S.Glasg., appointed House Surgeon to the Victoria Hospital for Children, Hull. D. Norman, M.D., appointed Professor of Pathological Anatomy at King's College, London. M. O'Driscoll, L.R.C.P., L.R.C.S.I., appointed Medical Officer for the Goleen Dispensary District. W. H. Pearce, M.R.C.S.Eng., appointed Medical Superintendent of the Poplar and Stepney District Sick Asylum. W. B. Pettit, L.R.C.P.Lond., M.R.C.S., appointed House Surgeon to the Worcester Infirmary. C. M. Phillips, L.R.C.P.,Lond., M.R.C.S., appointed Resident Medical Officer to the St. Pancras and Northern Dispensary. E. Riding, M.R.C.S.Eng., appointed Medical Officer for the Etwall District of the Burton-upon-Trent Union. E. M. Rodwell, M.R.C.S.Eng., L.S.A., appointed Medical Officer for the second District and of the Workhouse of the Loddon and Clavering Union. J. R. Russell, M.R.C.S., L.R.C.P., appointed House Surgeon to King's College Hospital. T. J. Selby, M.B.Durh., L.R.C.P., L.M., L.R.C.S.Edin., L.F.P.S.Glasg., appointed Deputy Medical Officer to the Runcorn Union Workhouse. H. C. Sharp, B.A., M.B., B.C.Cantab., appointed Resident Obstetric Surgeon to Guy's Hospital. T. W. Smith, L.S.A., appointed Physician Accoucheur's Assistant to King's College Hospital. W. R. Smith, M.B., appointed House Surgeon to King's College Hospital. Dr. W. B. Stamford, appointed Second Assistant Medical Officer at the Infirmary of the Shoreditch Union. W. M. Stevens, M.D.Lond., M.R.C.S., appointed Medical Tutor at the Bristol Medical School. W. Turner, M.R.C.S., L.R.C.P., appointed Assistant House Physician to King's College Hos-

MEDICAL VACANCIES AND APPOINTMENTS—(*continued*).

pital. M. A. Wade, L.R.C.P., L.M., L.R.C.S.I., appointed Medical Officer to the Collingwood Dock Bridewell, Liverpool. T. Warner, M.R.C.S., L.R.C.P., appointed Physician's Assistant to King's College Hospital. P. L. Webster, L.R.C.P., M.R.C.S., L.D.S., appointed Dental Surgeon to the Metropolitan Hospital. W. H. White, M.D., appointed Examiner in Materia Medica to the University of London. J. Whitehouse, F.R.C.S.Eng., appointed Honorary Surgeon to the Sunderland Infirmary. W. E. Williams, F.R.C.S.Edin., reappointed Medical Officer of Health for the Abertillery Urban Sanitary District. W. D. Wood, L.R.C.P., L.R.C.S.Edin., reappointed Medical Officer of Health for the Caversham Urban Sanitary District of the Henley Union.

### VACANCIES.

The following vacancies are announced this week, the amount of salary in each case being given after the name of the institution:

**Resident Medical Officers.**

Birmingham General Dispensary, Birmingham. Resident Surgeon for the Highgate Branch; doubly qualified. Salary £150 per annum, with allowance for cab hire, and furnished rooms, fire, lights, and attendance. Applications to Alex. Forrest, Secretary, by May 17th.
Bristol General Hospital. House Surgeon; doubly qualified. Salary £120 per annum, with board, residence, &c. Applications to the Secretary before May 15th.
Halifax Infirmary and Dispensary. Assistant House Surgeon; unmarried; doubly qualified. Salary £50 per annum, with residence, board, and washing. Applications to O. Webster, Secretary, by May 17th.
Hospital for Sick Children, Great Ormond Street, W.C. House Surgeon; unmarried. Appointment for six months. Salary £20, with board and residence in the hospital. Applications to the Secretary by May 16th.
Manchester Hospital for Consumption and Diseases of the Chest and Throat, Bowdon, Cheshire. Resident Medical Officer. Salary £60 per annum, with board, apartments, and washing. Applications to C. W. Hunt, Secretary, by May 18th.
Royal Free Hospital, Gray's Inn Road. Junior Resident Medical Officer. Board and residence provided. Appointment for six months. Applications to the Secretary by May 15th.

**Non-Resident Medical Officers.**

National Dental Hospital, Great Portland Street, W. Assistant Dental Surgeon; Anæsthetist. Applications to the Secretary by May 15th.
Parish of Glenelg. Medical Officer for the Northern Division. Salary £95 per annum, with free house and garden. Applications to D. McLure, Inspector of Poor, Glenelg.

### PASS LIST.

**Royal College of Physicians of London.**

At an ordinary meeting of the Comitia on April 27th, the following gentlemen, having conformed to the by-laws and regulations, and passed the required examinations, were among the gentlemen admitted Licentiates of the College, namely: E. L. Adams, Guy's; M. Ashley, University College; W. E. Baker, St. George's and London; W. R. Barrett, Charing Cross; H. S. Beadles, University College; T. W. Beazeley, Birmingham; D. M. Beddoe, Guy's; *R. T. Bedford, Guy's; F. Belben, Cambridge and St. Bartholomew's; E. H. Beresford, University College and Bristol; R. S. Bernard, King's College; E. H. Bingley, St. Mary's; B. W. Bond, St. Thomas's; H. R. Bowtell, Charing Cross; H. H. Brind, St. Mary's; W. Broadbent, Cambridge and St. Mary's; E. Brown, Charing Cross; T. F. Budden, Cambridge and University College; W. Butement, Otago and London; J. B. Byles, University College; E. S. Cardell, St. Bartholomew's; R. H. Castellote, University College; R. H. Chilton, Bristol; J. C. Clemesha, McGill; S. A. Coad, Leeds and St. Mary's; A. H. Conder, London; B. Cooper, St. George's; H. Cooper, Guy's; G. A. Copland, Otago and London; W. M. Cox, Birmingham; C. R. H. Crawford, St. Bartholomew's; R. H. Crowley, St. Bartholomew's; C. W. Curtis, Middlesex; A. D. Davies, London; J. N. D'Esterre, St. Mary's; H. M. Dowler, Westminster; H. D. Eccles, Guy's; W. A. Eden, King's College; G. Elliott, University College; A. V. Evans, St. George's; C. H. Evans, Cambridge and St. Bartholomew's; J. M. Evans, Glasgow and London; F. J. Fielder, King's College; J. H. Fletcher, Manchester; * F. J. Fletcher, Birmingham; G. A. T. Fox, St. Bartholomew's; I. George, King's College; W. H. Gossage, Westminster; W. R. Hadwen, Bristol and St. Bartholomew's; R. G. Hann, Leeds; *A. B. Harman, King's College; W. J. Harris, Cambridge and Guy's; F. Hazell, Guy's; L. G. Hill, London; J. O. Hollick, Birmingham and Durham; G. F. Holt, St. Bartholomew's; J. E. Horrocks, Manchester; M. J. Houghton, Birmingham and Middlesex; H. W. Hull, London; T. S. Jackson, Guy's; J. D. Jenkins, London; W. J. Johnson, Guy's; C. L. Jones, London; H. M. Jones, King's College; G. M. Keevil, Middlesex; A. E. Kennedy, London; J. Kennedy, London; G. T. Kevern, Bristol; L. Kilroy, Manchester and King's College; R. Knox, Edinburgh; J. Kyffin, London; C. A. Lapthorn, Middlesex; P. G. Laver, St. Thomas's.

* Candidates who have not presented themselves under the regulations of the Examining Board.

**ROYAL FREE HOSPITAL, Gray's Inn Road.**

**BIRMINGHAM GENERAL DISPENSARY.**

RESIDENT SURGEON WANTED for the Highgate Branch.

Candidates must be registered and possess both a Medical and a Surgical qualification.

Applications, together with ORIGINAL TESTIMONIALS AND CERTIFICATES OF REGISTRATION, must be forwarded to the undersigned on or before WEDNESDAY, May 17th.

Salary £150 per annum, with an allowance for cab hire, and Furnished Rooms, Fire, Lights, and Attendance.

(319) ALEX. FORREST, Secretary.

**THE HOSPITAL FOR SICK CHILDREN,**
Great Ormond Street, W.C.

